# Supplementary material for: Insight into binding of endogenous neurosteroid ligands to the sigma-1 receptor
Source: Nat Commun. 2024 Jul 4;15:5619. doi: 10.1038/s41467-024-49894-7 (PMC11224282; doi:10.1038/s41467-024-49894-7)
Supplement: Supplementary file 1 — Supplementary Information [file 41467_2024_49894_MOESM1_ESM.pdf]

Supplementary Information for

Insight into binding of endogenous neurosteroid ligands to the sigma-1 receptor

Chunting Fu[1‡], Yang Xiao[1‡], Xiaoming Zhou[1]*, Ziyi Sun[1]*

[1]Department of Integrated Traditional Chinese and Western Medicine, State Key Laboratory of Biotherapy, West China Hospital, Sichuan University, Chengdu, Sichuan 610041, China

[‡]These authors contributed equally to this work.

*To whom correspondence should be addressed:

Xiaoming Zhou, PhD, 17 Renmin Road South 3rd Section, Sichuan University, Chengdu, Sichuan 610041, China; Email: x.zhou@scu.edu.cn

Ziyi Sun, PhD, 17 Renmin Road South 3rd Section, Sichuan University, Chengdu, Sichuan 610041, China; Email: ziyi.sun@scu.edu.cn

Supplementary figures and tables

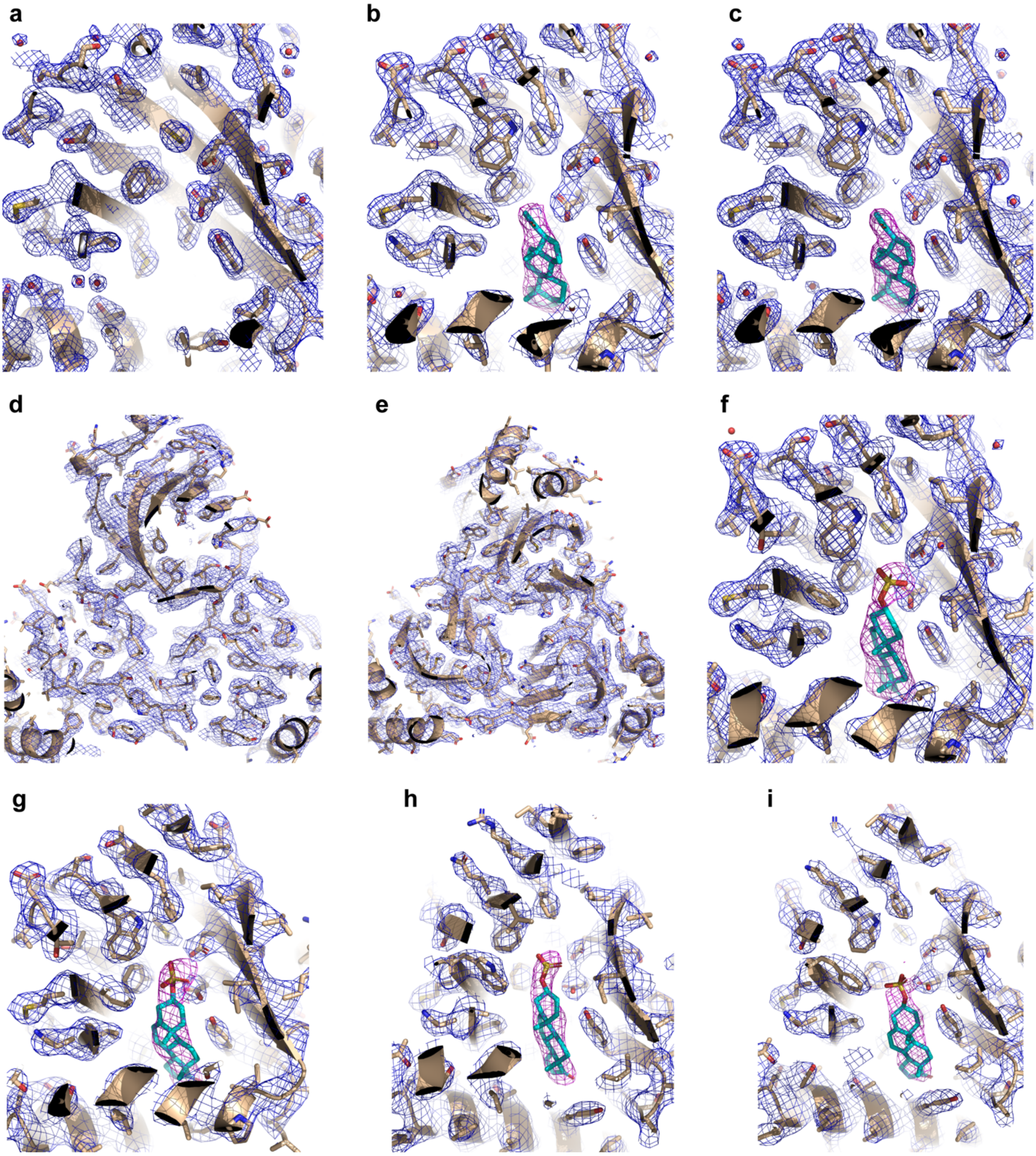

Supplementary Figure 1. Electron density maps of xlσ1R structures generated in this study. (a) xlσ1R$_{unknown-lig}$ (PDB ID: 8W4D). (b) xlσ1R$_{prog-soak}$ (PDB ID: 8W4C). (c) xlσ1R$_{prog-co}$ (PDB ID: 8W4B). (d) xlσ1R$_{side-open}$ (PDB ID: 8W4E). (e) xlσ1R$_{side-open-all}$ (PDB ID: 8YBB). (f) xlσ1R$_{DHEAS-I432}$ (PDB ID: 8WWB). (g)-(i) xlσ1R$_{DHEAS-C2}$ (PDB ID: 8WUE) protomer A (Pose-1, panel g), protomer B (Pose-2, panel h) and protomer C (Pose-3, panel i). In all panels, xlσ1R structures are shown in light orange, and the blue mesh shows the simulated annealing 2F$_o$-F$_c$ map contoured at 1.2 σ level.

In panels a, b, c and f, water molecules are shown as red spheres. In panels b, c, f, g, h and i, progesterone (panels b and c) or DHEAS (panels f, g, h and i) are shown as cyan sticks, and the purple mesh shows the simulated annealing F_o-F_c omit map contoured at 3.0 σ level.

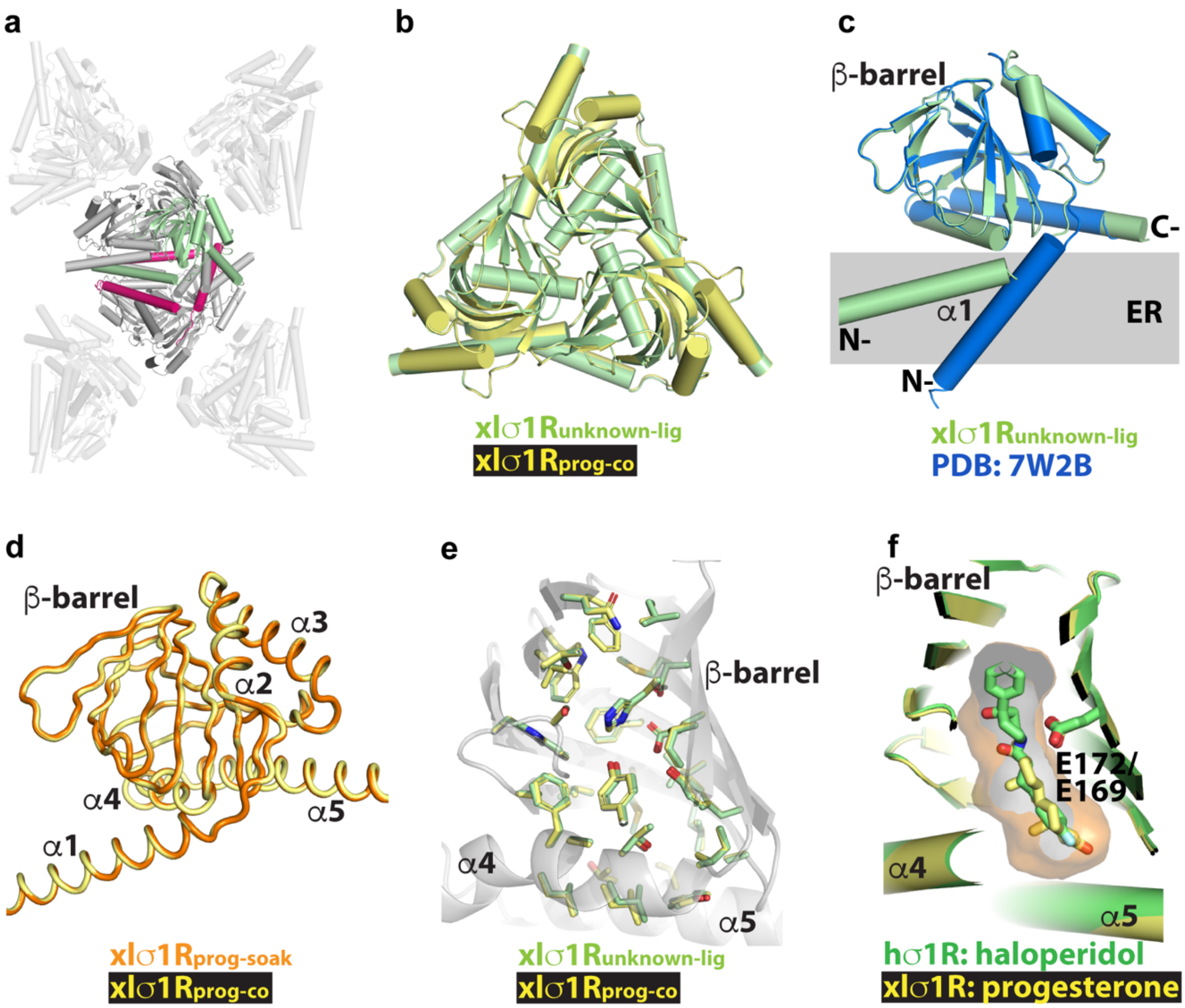

Supplementary Figure 2. Structural features of xlσ1R$_{unknown\text{-}lig}$ and the xlσ1R-progesterone complex. (a) Crystal packing of xlσ1R$_{unknown\text{-}lig}$ in the *I*432 space group. Each asymmetric unit contains one xlσ1R protomer (green cartoon). The α1 helices of a xlσ1R trimer are shown as red cylinders. (b) Superposition of the xlσ1R$_{prog\text{-}co}$ trimer (yellow cartoon) and the xlσ1R$_{unknown\text{-}lig}$ trimer (green cartoon). Viewed perpendicular to the membrane from the β-barrel side. (c) Superposition of xlσ1R$_{unknown\text{-}lig}$ (green cartoon) onto a previously reported xlσ1R structure (xlσ1R$_{closed\text{-}endo}$, PDB entry 7W2B, blue cartoon). The relative position of the ER membrane is indicated by a grey rectangle. (d) Superposition of a xlσ1R$_{prog\text{-}soak}$ protomer (orange tube) onto xlσ1R$_{prog\text{-}co}$ (yellow tube). (e) Superposition of lumen-lining residues between xlσ1R$_{unknown\text{-}lig}$ (green sticks) and xlσ1R$_{prog\text{-}co}$ (yellow sticks). (f) Superposition of xlσ1R$_{prog\text{-}co}$ (in yellow) onto the hσ1R-haloperidol structure (in lime green, PDB entry 6DJZ). Progesterone, haloperidol and residue E172/E169 are shown as sticks. The β-barrel lumen is indicated by orange surface.

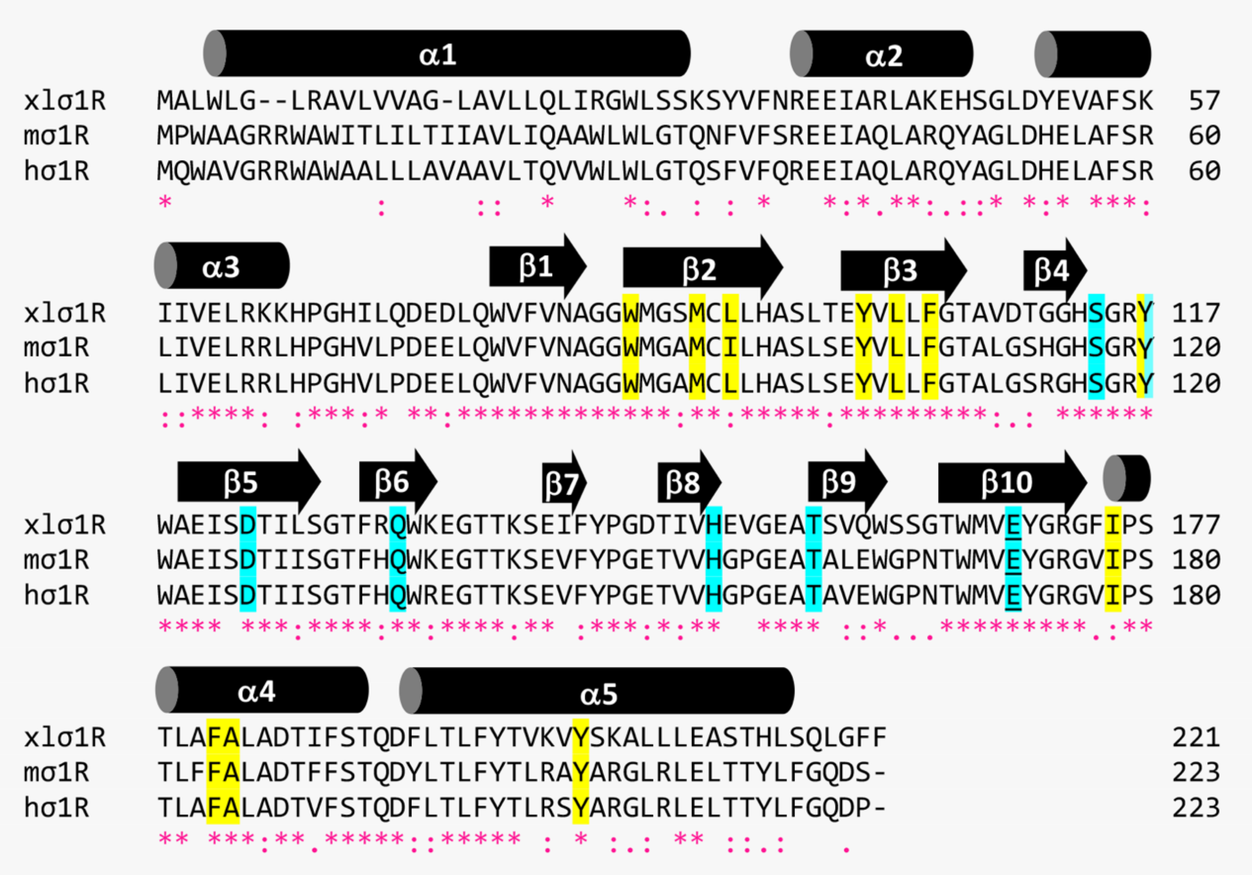

Supplementary Figure 3. Sequence alignment of σ1R homologs by ClustalW[1,2]. Alpha helices are indicated by cylinders and labeled from α1 to α5. The β-barrel is indicated by ten arrows labeled from β1 to β10. Residues participating in polar or hydrophobic interactions with ligands are highlighted in cyan or yellow, respectively. Residue Y117 is highlighted in both cyan and yellow to reflect that it may participate in both polar and hydrophobic interactions. The highly conserved residue E169 (xlσ1R)/E172 (hσ1R) is underscored. Asterisks (*) indicate identical residues. Colons (:) indicate strong similarities. Periods (.) indicate weak similarities. xlσ1R, σ1R from *Xenopus laevis*; mσ1R, σ1R from *Mus musculus*; hσ1R, σ1R from *Homo sapiens*.

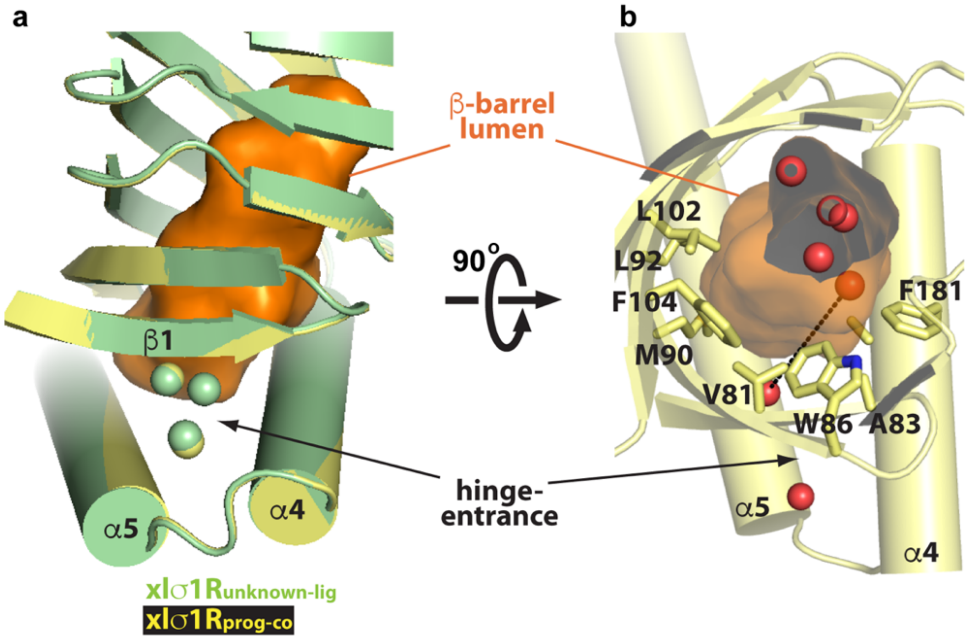

Supplementary Figure 4. Water near the hinge-entrance of xlσ1R. (a) Water molecules (yellow/green spheres) near the hinge-entrance of xlσ1R$_{prog\text{-}co}$ (yellow cartoon) and xlσ1R$_{unknown\text{-}lig}$ (green cartoon). (b) The shortest distance between the hinge-entrance water (red spheres) and the distal β-barrel lumen water (red spheres) in xlσ1R$_{prog\text{-}co}$ is indicated by a black dashed line. Residues along the line are shown as sticks. In both panels, only the β-barrel and α4/α5 of xlσ1R are shown for clearer views.

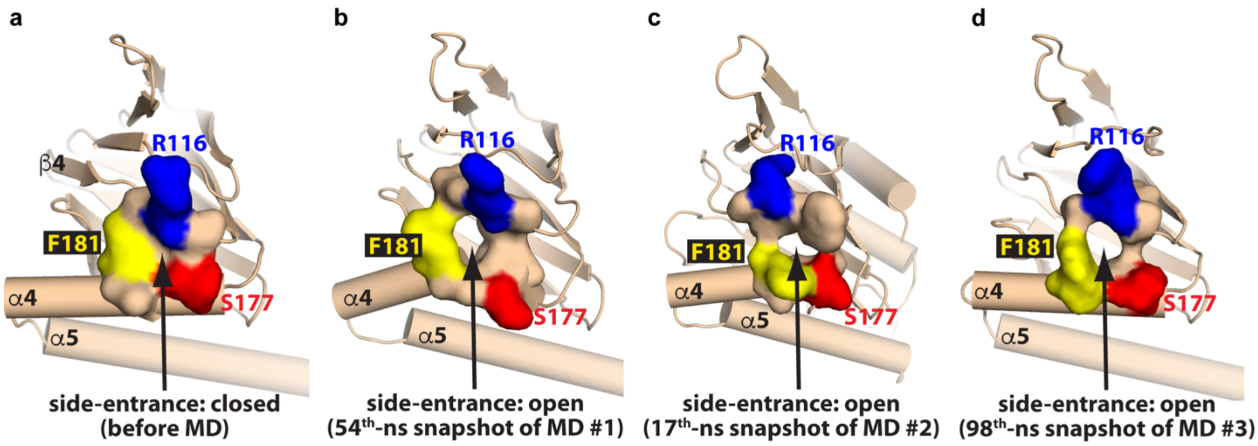

Supplementary Figure 5. MD simulation of xlσ1R$_{prog\text{-}co}$. (a) Snapshot of a xlσ1R$_{prog\text{-}co}$ monomer showing a closed side-entrance before MD simulation. (b)-(d) Snapshots of xlσ1R$_{prog\text{-}co}$ showing an open side-entrance at 54th ns of MD run #1 (panel b), 17th ns of MD run #2 (panel c) and 98th ns of MD run #3 (panel d). The location of the side-entrance is indicated by a black arrow. Residues surrounding the side-entrance are displayed in surface mode. Among these residues, R116 (β4/β5 loop), S177 (α4) and F181 (α4) are colored in blue, red and yellow, respectively. In all panels, only the β-barrel and α4/α5 of xlσ1R are shown for clearer views.

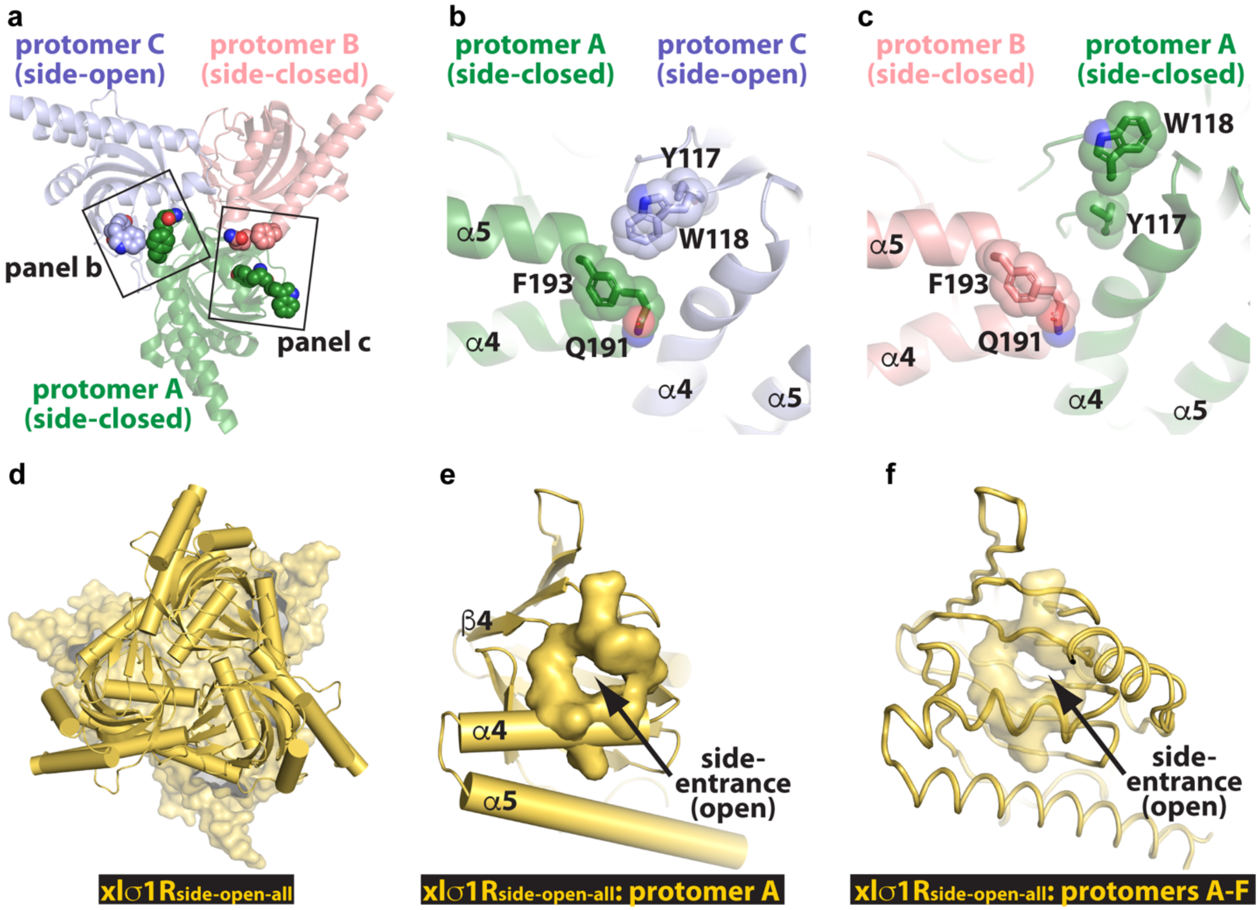

Supplementary Figure 6. The side-open conformation of xlσ1R structures. (a) A trimer of the xlσ1R$_{side\text{-}open}$ structure. Three protomers (A, B and C) are shown in different colors. Residues Y117 and W118 of one protomer and residues Q191 and F193 of the adjacent protomer are rendered in spheres. For a clearer view, α1 is not shown in this panel. (b) A close-up view of panel a, showing the contacting region between protomer A (side-closed, in green) and protomer C (side-open, in light blue). (c) A close-up view of panel a, showing the contacting region between protomer A (side-closed, in green) and protomer B (side-closed, in pink). (d) Structure of xlσ1R$_{side\text{-}open\text{-}all}$ with each asymmetric unit containing two trimers (six protomers) in yellow orange. (e) Protomer A of xlσ1R$_{side\text{-}open\text{-}all}$. (f) Superposition of protomers A to F (all in yellow orange) of xlσ1R$_{side\text{-}open\text{-}all}$. In panels e and f, residues 114-118 (β4/β5 loop) and 177-181 (α4) are displayed in surface mode, and the location of the side-entrance is indicated by a black arrow. In panels e and f, only the β-barrel and α4/α5 of xlσ1R are shown for clearer views.

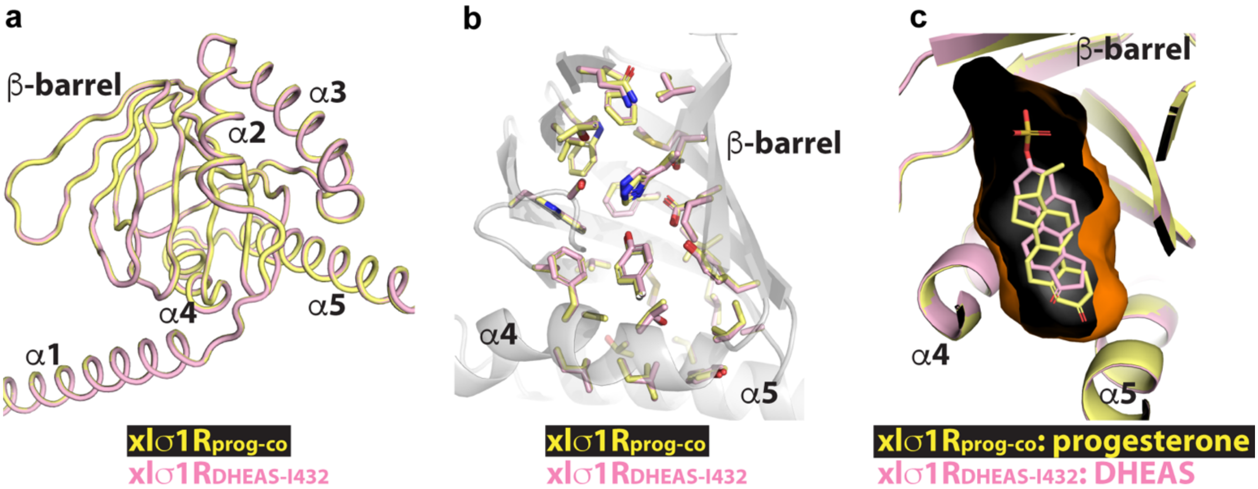

Supplementary Figure 7. Comparison between xlσ1R$_{prog\text{-}co}$ and xlσ1R$_{DHEAS\text{-}I432}$. (a) Superposition of xlσ1R$_{DHEAS\text{-}I432}$ (pink tube) onto xlσ1R$_{prog\text{-}co}$ (yellow tube). (b) Superposition of lumen-lining residues between xlσ1R$_{DHEAS\text{-}I432}$ (pink sticks) and xlσ1R$_{prog\text{-}co}$ (yellow sticks). (c) Comparison of bound progesterone (yellow sticks) to DHEAS (pink sticks) in the β-barrel lumen (orange surface) of xlσ1R$_{prog\text{-}co}$ (yellow cartoon) and xlσ1R$_{DHEAS\text{-}I432}$ (pink cartoon), respectively.

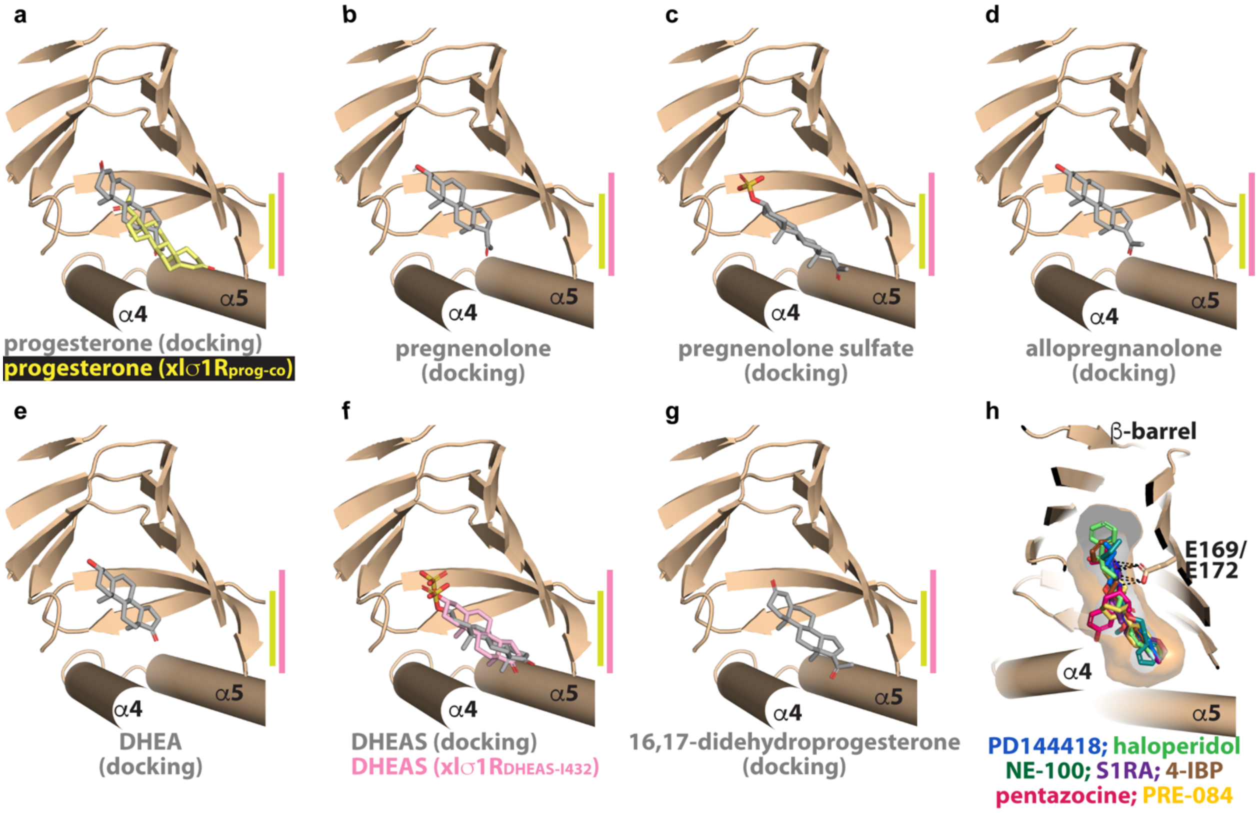

Supplementary Figure 8. Docking of potential steroid ligands into xlσ1R. (a)-(g) The top-scored docking models of progesterone (panel a), pregnenolone (panel b), pregnenolone sulfate (panel c), allopregnanolone (panel d), DHEA (panel e), DHEAS (panel f) and 16,17-didehydroprogesterone (panel g) using the protein portion of the xlσ1R$_{prog\text{-}co}$ structure (see Methods). Docking models of all steroids are displayed as grey sticks. Progesterone and DHEAS from the xlσ1R$_{prog\text{-}co}$ and xlσ1R$_{DHEAS\text{-}I432}$ structures are shown as yellow (panel a) and pink sticks (panel f), respectively, and their binding locations are indicated by a yellow bar and a pink bar, respectively, on the right of each panel. (h) Published structures of hσ1R/ xlσ1R bound to synthetic ligands, which are shown as color sticks. Potential polar interactions between the basic nitrogen atom of ligands and the E169/E172 side chain of σ1R are indicated by black dashed lines. In all panels, only the β-barrel and α4/α5 of xlσ1R are shown for clearer views.

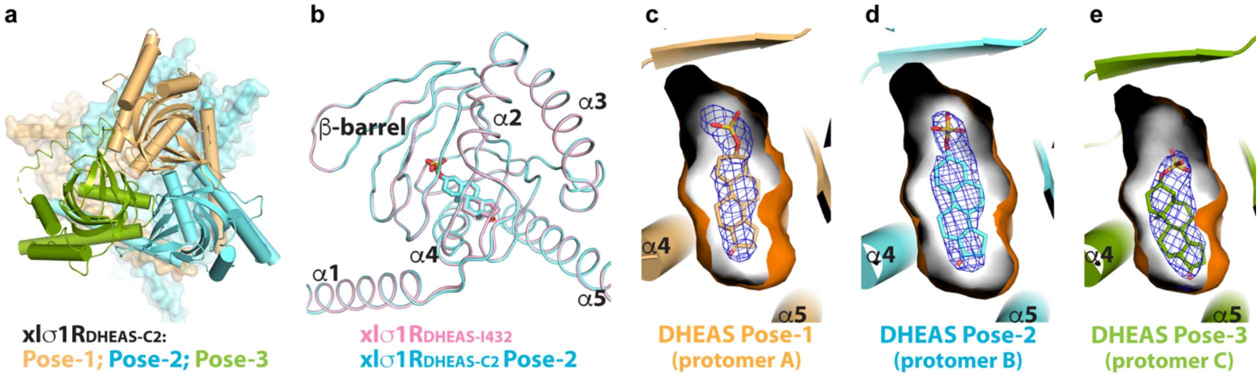

Supplementary Figure 9. Structure of xlσ1R$_{DHEAS-C2}$. (a) Six protomers in each asymmetric unit of xlσ1R$_{DHEAS-C2}$ with three different DHEAS binding poses. DHEAS Pose-1 in protomers A and D (light orange cartoon); DHEAS Pose-2 in protomers B, E and F (cyan cartoon); DHEAS Pose-3 in protomer C (green cartoon). (b) Superposition of xlσ1R$_{DHEAS-C2}$ Pose-2 (in cyan) onto xlσ1R$_{DHEAS-I432}$ (in pink). DHEAS is shown as sticks. (c)-(e) Density fitting of DHEAS Pose-1 (light orange sticks, panel c), Pose-2 (cyan sticks, panel d), and Pose-3 (green sticks, panel e) in the β-barrel lumen (orange surface) of xlσ1R$_{DHEAS-C2}$ protomer A (panel c), protomer B (panel d), and protomer C (panel e). In panels c, d and e, the blue mesh shows the simulated annealing 2F$_o$-F$_c$ map contoured at 1.2 σ level.

Supplementary Table 1. Published hσ1R/ xlσ1R structures bound to synthetic ligands.

σ1R source	Ligand name	Ligand class	Chemical structure*	PDB ID
	PD144418	Antagonist	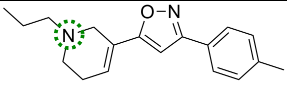	5HK1
	4-IBP	Unknown	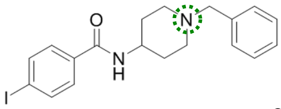	5HK2
Homo sapiens (hσ1R)	haloperidol	Antagonist	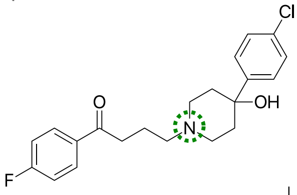	6DJZ
	NE-100	Antagonist	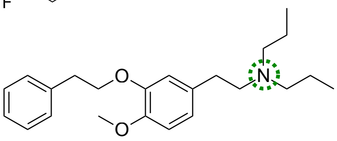	6DK0
	(+)-pentazocine	Agonist	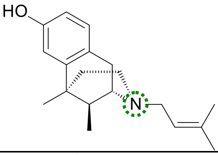	6DK1
Xenopus laevis (xlσ1R)	PRE-084	Agonist	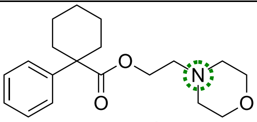	7W2C, 7W2F, 7W2G
	S1RA	Antagonist	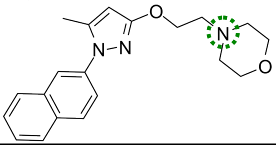	7W2D

*The basic nitrogen atom that interacts with E169 (xlσ1R) or E172 (hσ1R) through direct polar interactions is indicated by a green dashed circle.

Supplementary Table 2. Potential steroid ligands for σ1R.

Steroid name	Molecular formula	Chemical structure	PubChem ID
progesterone	$C_{21}H_{30}O_2$	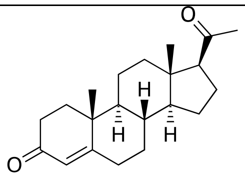	5994
pregnenolone	$C_{21}H_{32}O_2$	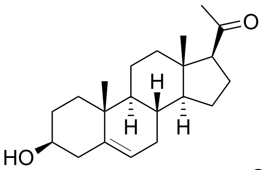	8955
pregnenolone sulfate	$C_{21}H_{32}O_5S$	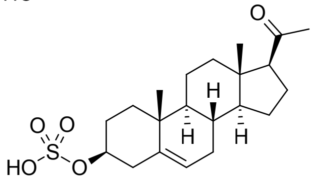	105074
allopregnanolone	$C_{21}H_{34}O_2$	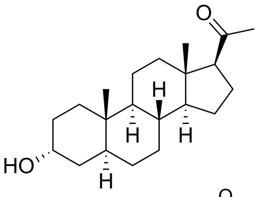	92786
dehydroepiandrosterone (DHEA)	$C_{19}H_{28}O_2$	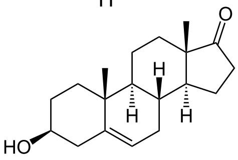	5881
dehydroepiandrosterone sulfate (DHEAS)	$C_{19}H_{28}O_5S$	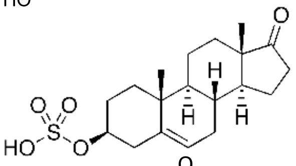	12594
16,17-didehydroprogesterone	$C_{21}H_{28}O_2$	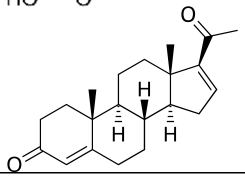	101964

Supplementary Table 3. Affinities (K_d) of two neurosteroid ligands to xlσ1R determined by MST.

Neurosteroid Ligand	Receptor (xlσ1R)	Repeat 1 (μM)	Repeat 2 (μM)	Repeat 3 (μM)	Mean (μM)	SD (μM)	*P*
Progesterone	Wild-type	0.71	0.96	1.14	0.94	0.22	-
	E169A	0.92	1.19	0.85	0.98	0.18	0.78
DHEAS	Wild-type	17.2	15.9	20.8	18.0	2.5	-
	E169A	470.9	437.6	460.0	456.2	17.0	0.0000016

MST measurements were repeated with N=3 biologically independent samples and source data of K_d values are shown. Two-tailed Student's t-test was performed between wild-type and E169A for each ligand, and the P values are shown.

Supplementary Table 4. Interaction/binding energies between xlσ1R and bound neurosteroids.

Receptor	Interaction energy (AMMOS2 web server)		Binding energy (BIOVIA Discovery Studio)	
	Progesterone (kcal/mol)	DHEAS (kcal/mol)	Progesterone (kcal/mol)	DHEAS (kcal/mol)
xlσ1R$_{prog\text{-}co}$: 0 water*	-58.6	-	-48.8	-
xlσ1R$_{prog\text{-}co}$: 1 water* (Water403)	-59.6	-	-	-
xlσ1R$_{prog\text{-}co}$: 6 water*	-62.0	-	-53.0	-
xlσ1R$_{DHEAS\text{-}I432}$: 0 water*	-	-30.7	-	-43.5
xlσ1R$_{DHEAS\text{-}I432}$: 2 water*	-	-30.9	-	-45.6
xlσ1R$_{DHEAS\text{-}C2}$ protomer A (Pose-1)	-	-48.4	-	-
xlσ1R$_{DHEAS\text{-}C2}$ protomer B (Pose-2)	-	-24.3	-	-
xlσ1R$_{DHEAS\text{-}C2}$ protomer C (Pose-3)	-	-49.7	-	-

*Water refers to the water molecules within the distal space of the β-barrel lumen of xlσ1R structures (See Methods).

Supplementary Table 5. Molecular dynamics simulations checklist.

**Reliability and reproducibility checklist for molecular dynamics simulations** ***All boxes must be marked YES by acceptance unless an N/A option is available**	**Yes**	**N/A**	**Response (Please state where this information can be found in the text)**
**1. Convergence of simulations and analysis**			
1a. Is an evaluation presented in the text to show that the property being measured has equilibrated in the simulations (*e.g.* time-course analysis)?	☒		The system was equilibrated for 125 ps using a NVT ensemble (constant Number of particles, Volume, and Temperature), followed by a 125-ps NPT equilibration (constant Number of particles, Pressure, and Temperature), while the protein and crystallographic water molecules were fixed. The system was well-equilibrated as indicated by reaching stable temperature, pressure and density over time before production run. **This information can be found in the Methods section.**
1b. Then, is it described in the text how simulations are split into equilibration and production runs and how much data were analyzed from production runs?	☒		Production runs (100 ns) were conducted after equilibration runs (125-ps NVT + 125-ps NPT) had been completed. 100 snapshots from each production run (1 snapshot per ns for 100 ns) were analyzed. **This information can be found in the Methods section.**
1c. Are there at least 3 simulations per simulation condition with statistical analysis?	☒		Three parallel runs (100 ns each) were conducted without positional restraints at a temperature of 303 K and a constant pressure of 1 bar, and RMSD of the protein backbone from its initial to final states was utilized to analyze convergence of simulations. **This information can be found in the Methods section.**
1d. Is evidence provided in the text that the simulation results presented are independent of initial configuration?	☒		Simulations were conducted with different initial velocities, and similar side-entrances have been observed in all simulations. **This information can be found in the Methods section.**
**2. Connection to experiments**			
2a. Are calculations provided that can connect to experiments (*e.g.* loss or gain in function from mutagenesis, binding assays, NMR chemical shifts, J-couplings, SAXS curves, interaction distances or FRET distances, structure factors, diffusion coefficients, bulk modulus and other mechanical properties, *etc.*)?	☒		The goal of simulations in this study is to qualitatively identify an opening(s) on the protein surface as a potential water entrance(s). This observation connects to , and is supported by experimental structures in this study. No calculation or quantitative measurement was conducted with simulation results. **This**

			**information can be found in the Results section.**
**3. Method choice**			
3a. Is it described in the text what force field and water model are used and why?	☒		CHARMM36m force field and TIP3P water model were used, which are common and effective choice in GROMACS. **This information can be found in the Methods section.**
3b. Do simulations contain membranes, membrane proteins, intrinsically disordered proteins, glycans, nucleic acids, polymers, or cryptic ligand binding?	☐	☒	Response not needed if **N/A**
If 3b is **YES**, are enhanced sampling methods used?	☐	☒	Response not needed if **N/A**
If enhanced sampling methods are used, are the convergence criteria clearly stated?	☐		**N/A**
If 3b is **YES**, is it explained in the text why or why not enhanced sampling methods are used?	☐		**N/A**
**4. Code and reproducibility**			
4a. Is a table provided describing the system setup, such as simulation box dimensions, total number of atoms, total number of water molecules, salt concentration, lipid composition (number of molecules and type)?	☒		The final simulation box (8.2 nm × 8.2 nm × 8.2 nm) contains a total of 51,745 atoms, including xlσ1R$_{prog\text{-}co}$ (residues 34-219), 16,247 water molecules, 52 K^+ and 46 Cl^-. **This information can be found in the Methods section.**
4b. Is it described in the text what simulation and analysis software and which versions are used?	☒		Simulation software: GROMACS (BUILT 2023.2);<br>Analysis software: UCSF ChimeraX v1.4. **This information can be found in the Methods section.**
4c. Are initial coordinate and simulation input files and a coordinate file of the final output provided as supplementary files or in a public repository?	☒		The initial input coordinate file (Supplementary Data 5) and final output coordinate files (Supplementary Data 6-8) of simulations have been provided as Supplementary Data files.
4d. Is there custom code or custom force field parameters?	☐	☒	Response not needed if **N/A**
If **YES**, are they provided as supplementary profiles or in a public repository?	☐		**N/A**

Supplementary References

1. Thompson, J.D., Higgins, D.G. & Gibson, T.J. CLUSTAL W: improving the sensitivity of progressive multiple sequence alignment through sequence weighting, position-specific gap penalties and weight matrix choice. *Nucleic Acids Res* **22**, 4673-80 (1994).
2. Combet, C., Blanchet, C., Geourjon, C. & Deleage, G. NPS@: network protein sequence analysis. *Trends Biochem Sci* **25**, 147-50 (2000).
