# Supplementary material for: Insight into binding of endogenous neurosteroid ligands to the sigma-1 receptor
Source: Nat Commun. 2024 Jul 4;15:5619. doi: 10.1038/s41467-024-49894-7 (PMC11224282; doi:10.1038/s41467-024-49894-7)
Supplement: Supplementary file 3 — Description of Additional Supplementary Files [file 41467_2024_49894_MOESM3_ESM.pdf]

Description of Additional supplementary file

Supplementary Data 1. binding-energy_8W4B-0water-progesterone.

Supplementary Data 2. binding-energy_8W4B-6water-progesterone.

Supplementary Data 3. binding-energy_8WWB-0water-DHEAS.

Supplementary Data 4. binding-energy_8WWB-2water-DHEAS.

Supplementary Data 5. MD-input initial-coordinate.

Supplementary Data 6. MD-run01-output_final-coordinate.

Supplementary Data 7. MD-run02-output_final-coordinate.

Supplementary Data 8. MD-run03-output_final-coordinate.

Supplementary Data 9. dock progesterone.

Supplementary Data 10. dock pregnenolone.

Supplementary Data 11. dock_ pregnenolone sulfate.

Supplementary Data 12. dock allopregnanolone.

Supplementary Data 13. Deck_head.

Supplementary Data 14. Deck_heads.

Supplementary Data 15. dock_16, 17-didehydroprogesterone.
