# Supplementary material for: Insight into binding of endogenous neurosteroid ligands to the sigma-1 receptor
Source: Nat Commun. 2024 Jul 4;15:5619. doi: 10.1038/s41467-024-49894-7 (PMC11224282; doi:10.1038/s41467-024-49894-7)
Supplement: Supplementary file 19 — Reporting Summary [file 41467_2024_49894_MOESM19_ESM.pdf]

nature portfolio

Corresponding author(s): Xiaoming Zhou, Ziyi Sun

Last updated by author(s): Jun 3, 2024

Reporting Summary

Nature Portfolio wishes to improve the reproducibility of the work that we publish. This form provides structure for consistency and transparency in reporting. For further information on Nature Portfolio policies, see our Editorial Policies and the Editorial Policy Checklist.

Statistics

For all statistical analyses, confirm that the following items are present in the figure legend, table legend, main text, or Methods section.

n/a	Confirmed	
	x	The exact sample size (*n*) for each experimental group/condition, given as a discrete number and unit of measurement
	x	A statement on whether measurements were taken from distinct samples or whether the same sample was measured repeatedly
	x	The statistical test(s) used AND whether they are one- or two-sided<br>*Only common tests should be described solely by name; describe more complex techniques in the Methods section.*
x		A description of all covariates tested
x		A description of any assumptions or corrections, such as tests of normality and adjustment for multiple comparisons
	x	A full description of the statistical parameters including central tendency (e.g. means) or other basic estimates (e.g. regression coefficient) AND variation (e.g. standard deviation) or associated estimates of uncertainty (e.g. confidence intervals)
	x	For null hypothesis testing, the test statistic (e.g. *F*, *t*, *r*) with confidence intervals, effect sizes, degrees of freedom and *P* value noted<br>*Give P values as exact values whenever suitable.*
x		For Bayesian analysis, information on the choice of priors and Markov chain Monte Carlo settings
x		For hierarchical and complex designs, identification of the appropriate level for tests and full reporting of outcomes
x		Estimates of effect sizes (e.g. Cohen's *d*, Pearson's *r*), indicating how they were calculated

Our web collection on statistics for biologists contains articles on many of the points above.

Software and code

Policy information about availability of computer code

Data collection	For MST measurement: MO.Control v1.6.1.
Data analysis	For X-ray diffraction data processing: autoPROC pipeline package 1.0.5, XDS BUILT 20230630, CCP4 v7.0.072.<br>For phasing, model building and display: Phaser 2.8.1, Phenix 1.13-2998 (containing phenix.refine and phenix.table_one), COOT 0.8.9.1, MacPyMOL (PyMOL v1.8.0.6 Enhanced for Mac OS X), HOLLOW 1.1, PROPKA 3.1.<br>For interaction energy calculation: AMMOS2 web server (http://drugmod.rpbs.univ-paris-diderot.fr/ammosHome.php).<br>For binding energy calculation: BIOVIA Discovery Studio 2021.<br>For MD simulation: GROMACS BUILT 2023.2, CHARMM-GUI web server (https://charmm-gui.org), UCSF ChimeraX 1.4.<br>For molecular docking: AutoDock Vina 1.1.2, MGLTools 1.5.6 (containing AutoDock Tools), UCSF Chimera 1.15.<br>For MST binding analysis: MO.Affinity Analysis v2.2.4, Microsoft Excel for Mac 2016.

For manuscripts utilizing custom algorithms or software that are central to the research but not yet described in published literature, software must be made available to editors and reviewers. We strongly encourage code deposition in a community repository (e.g. GitHub). See the Nature Portfolio guidelines for submitting code & software for further information.

Data

Policy information about availability of data

All manuscripts must include a data availability statement. This statement should provide the following information, where applicable:

- Accession codes, unique identifiers, or web links for publicly available datasets
- A description of any restrictions on data availability
- For clinical datasets or third party data, please ensure that the statement adheres to our policy

The atomic coordinates and structure factors of the xlS1R structures generated in this study have been deposited in the Protein Data Bank under the following accession codes: 8W4B (xlS1Rprog-co) [https://doi.org/10.2210/pdb8W4B/pdb], 8W4C (xlS1Rprog-soak) [https://doi.org/10.2210/pdb8W4C/pdb], 8W4D (xlS1Runknown-lig) [https://doi.org/10.2210/pdb8W4D/pdb], 8WWB (xlS1RDHEAS-I432) [https://doi.org/10.2210/pdb8WWB/pdb], 8WUE (xlS1RDHEAS-C2) [https://doi.org/10.2210/pdb8WUE/pdb], 8W4E (xlS1Rside-open) [https://doi.org/10.2210/pdb8W4E/pdb] and 8YBB (xlS1Rside-open-all) [https://doi.org/10.2210/pdb8YBB/pdb]. The MST data and interaction/binding energy values are provided in the Supplementary Information or Source Data file. Coordinates of optimized ligand models of progesterone or DHEAS from binding energy calculations, the initial input coordinate file and final output coordinate files of MD simulations, and docking models were provided as Supplementary Data files. Previously reported hS1R and xlS1R structures used in this study are available in the Protein Data Bank under the following accession codes: 5HK1 (hS1R: PD144418) [https://doi.org/10.2210/pdb5HK1/pdb], 5HK2 (hS1R: 4-IBP) [https://doi.org/10.2210/pdb5HK2/pdb], 6DJZ (hS1R: haloperidol) [https://doi.org/10.2210/pdb6DJZ/pdb], 6DK0 (hS1R: NE-100) [https://doi.org/10.2210/pdb6DK0/pdb], 6DK1 (hS1R: pentazocine) [https://doi.org/10.2210/pdb6DK1/pdb], 7W2B (xlS1Rclosed-endo) [https://doi.org/10.2210/pdb7W2B/pdb], 7W2C (xlS1Rclosed-PRE084) [https://doi.org/10.2210/pdb7W2C/pdb], 7W2D (xlS1Rclosed-S1RA) [https://doi.org/10.2210/pdb7W2D/pdb] and 7W2E (xlS1Ropen-endo) [https://doi.org/10.2210/pdb7W2E/pdb]. Source data are provided with this paper.

Research involving human participants, their data, or biological material

Policy information about studies with human participants or human data. See also policy information about sex, gender (identity/presentation), and sexual orientation and race, ethnicity and racism.

Reporting on sex and gender	Human participants or human data are not involved in this research.
Reporting on race, ethnicity, or other socially relevant groupings	Human participants or human data are not involved in this research.
Population characteristics	Human participants or human data are not involved in this research.
Recruitment	Human participants or human data are not involved in this research.
Ethics oversight	Human participants or human data are not involved in this research.

Note that full information on the approval of the study protocol must also be provided in the manuscript.

Field-specific reporting

Please select the one below that is the best fit for your research. If you are not sure, read the appropriate sections before making your selection.

[x] Life sciences [ ] Behavioural & social sciences [ ] Ecological, evolutionary & environmental sciences

For a reference copy of the document with all sections, see nature.com/documents/nr-reporting-summary-flat.pdf

Life sciences study design

All studies must disclose on these points even when the disclosure is negative.

Sample size	The sample size (N=3 biologically independent samples) was chosen from similar MST experiments from former publications, which is a commonly used sample size for MST measurements.
Data exclusions	No data was excluded from analysis.
Replication	All experiments were performed independently for 3 times. All attempts at replication were successful under the experimental conditions defined.
Randomization	Purified protein samples were randomly assigned to different neurosteroids.
Blinding	Protein samples were purified by multiple researchers, and MST measurements and analyses were performed by different researchers without knowing the protein identity.

Reporting for specific materials, systems and methods

We require information from authors about some types of materials, experimental systems and methods used in many studies. Here, indicate whether each material, system or method listed is relevant to your study. If you are not sure if a list item applies to your research, read the appropriate section before selecting a response.

Materials & experimental systems

n/a	Involved in the study
☒	☐ Antibodies
☒	☐ Eukaryotic cell lines
☒	☐ Palaeontology and archaeology
☒	☐ Animals and other organisms
☒	☐ Clinical data
☒	☐ Dual use research of concern
☒	☐ Plants

Methods

n/a	Involved in the study
☒	☐ ChIP-seq
☒	☐ Flow cytometry
☒	☐ MRI-based neuroimaging

Plants

Seed stocks	Plants are not involved in this research.
Novel plant genotypes	Plants are not involved in this research.
Authentication	Plants are not involved in this research.
